# Sclerotherapy of Giant Abdominal Lymphangiomas in a Six-Year-Old Boy: A Case Report and Literature Review

**DOI:** 10.7759/cureus.26914

**Published:** 2022-07-16

**Authors:** Medhat S Al-Sofyani, Njoud N Alharbi, Maram S Alotaibi, Razan M Alotaibi

**Affiliations:** 1 Pediatric Surgery, Security Forces Hospital, Riyadh, SAU; 2 Medicine, Princess Nourah Bint Abdulrahman University, Riyadh, SAU

**Keywords:** pediatric, sclerotherapy, doxycycline, lymphatic malformations, abdominal lymphangioma

## Abstract

Abdominal lymphangiomas are an uncommon form of congenital lymphatic malformation. We describe a case of a six-year-old male child who presented to our hospital with pain in the abdomen after an alleged history of minor trauma to the abdomen. On per abdomen examination there was a visible mass that was cystic on palpation.

Abdominal ultrasound and magnetic resonance imaging showed large multiloculated, septated intra-abdominal cystic structures. The patient underwent sclerotherapy with doxycycline and resolved completely after one session without complications. Doxycycline sclerotherapy is very effective and safe in treating macrocystic lymphatic malformations.

## Introduction

Lymphatic malformations (LMs) are vascular low-flow malformations as classified by the International Society for the Study of Vascular Anomalies (ISSVA) [1]. LMs are divided into three categories; the first occurs due to the lymphatic vessel and lymph node anomalies leading to impairment of lymphatic drainage resulting in lymphedema. The second category is varying-sized lymphatic cysts, and the third is central conducting lymphatic anomalies that relate to the circulation of chyle. LMs can be isolated or combined with other malformations of arteries, veins, and capillaries [1].

Abdominal LMs can be classified based on the anatomic region as purely intra-abdominal, mesenteric, retroperitoneal, and pelvic. Symptoms vary depending on the location, size, and extent of involvement of neighboring organs [2]. Abdominal LM reportedly constitutes anywhere from 3% to 9.2% of LMs in children, but the exact incidence is not known.

## Case presentation

A previously healthy six-year-old boy was admitted to ER in January 2021 with a three-day history of abdominal pain and distention after minor trauma. No fever, nausea, vomiting, constipation, or change in bowel habits were reported, and there was no significant past medical or surgical history.

**Figure 1:**
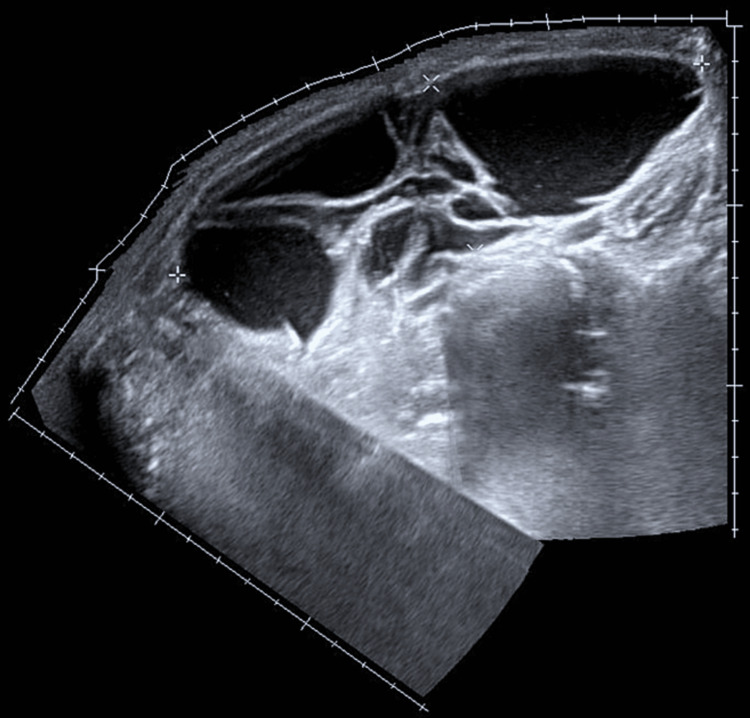
Sagittal abdominal ultrasound image demonstrating multiple large multiloculated, septated cystic structures.

**Figure 2:**
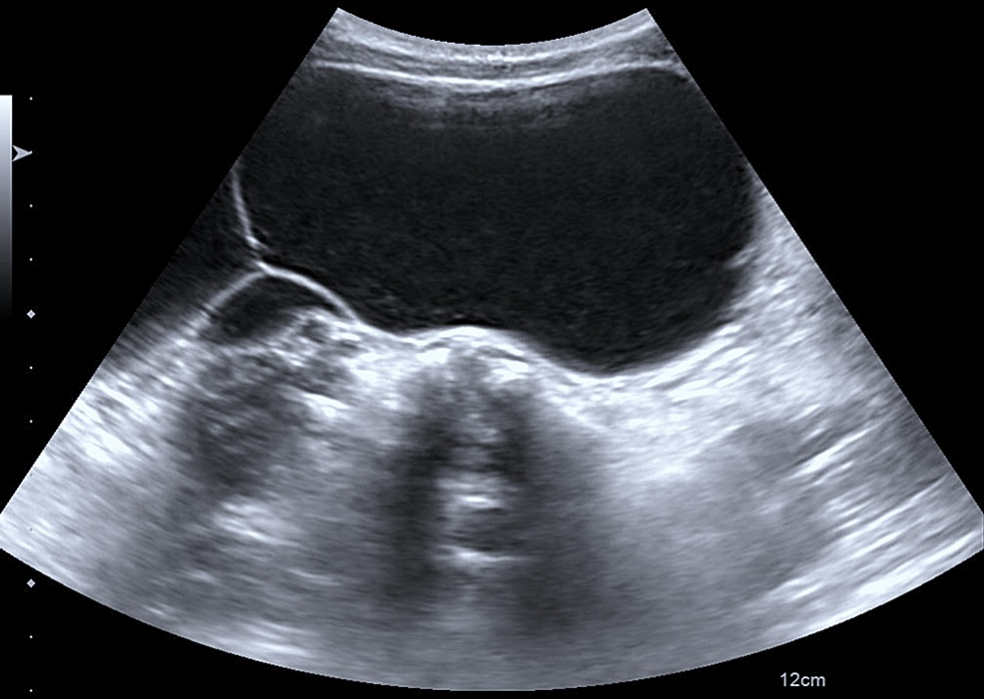
Transverse abdominal ultrasound image demonstrating multiple large multiloculated, septated cystic structures.

On physical examination, he looked well and comfortable with normal vital signs. The abdomen was distended in the left upper quadrant with a palpable tender cystic mass. Ultrasound of the abdomen showed multiple large multiloculated, septated cystic structures, the largest measuring 15.48 x 4.79 cm (Figures 1, 2).

**Figure 3:**
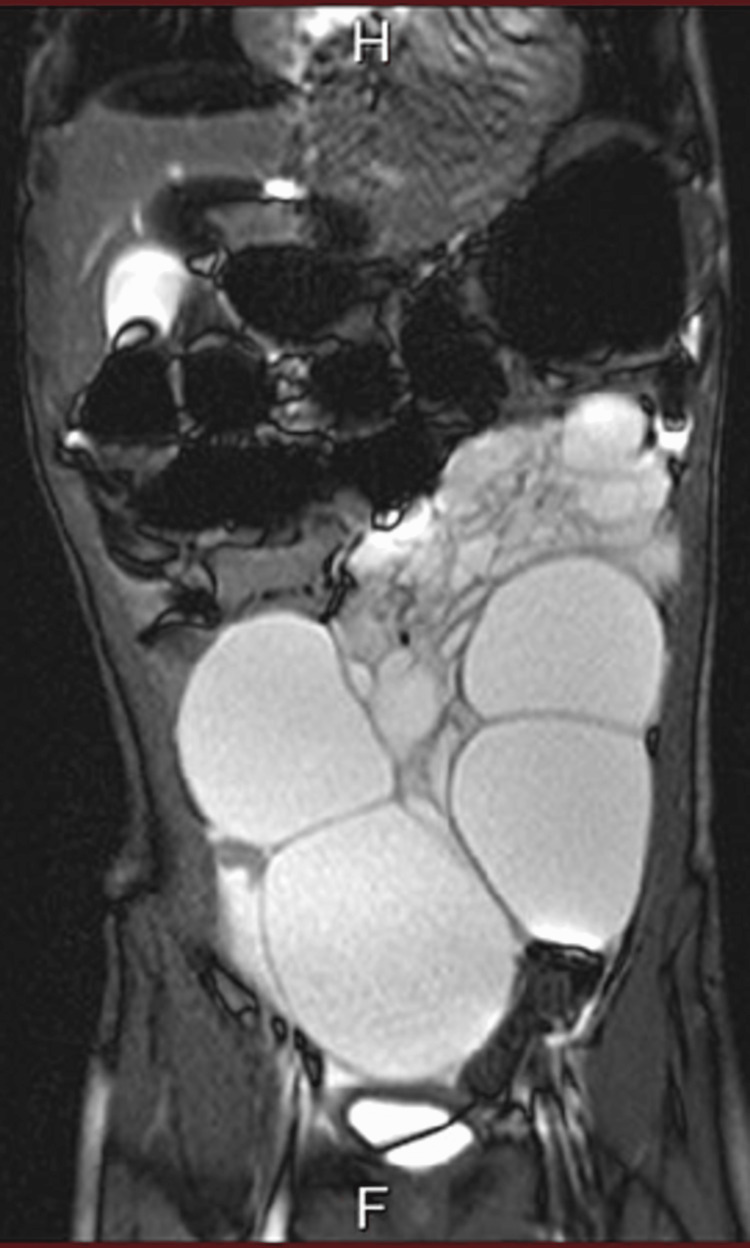
Coronal MRI of the abdomen shows cystic lesions filling the abdominal cavity and displacing the small bowel to the right upper quadrant. MRI: Magnetic resonance imaging

Magnetic resonance imaging (MRI) showed the same cystic lesions involving the abdominal cavity and displacing the small bowel to the right upper quadrant (Figure 3). The diagnosis of multilocular intra-abdominal lymphangiomas presenting after minor trauma was made.

The patient underwent ultrasound and fluoroscopy-guided sclerotherapy under GA and this procedure was done by an interventional radiologist. The cystic structures were targeted with an 18-gauge needle and then an 8 Fr. pigtail catheter was inserted followed by aspiration of the turbid serous content. Water-soluble contrast was injected to identify possible leak or communication with other structures, then the same amount of aspirated fluid was replaced with doxycycline mixture that was prepared to give a final concentration of 10 mg/mL. The total volume of doxycycline solution that was used was 300 mL after removing approximately 320 mL of fluid through three different pigtail catheters in three different cystic structures. There was no immediate complication. The patient had only one session of sclerotherapy and was discharged home on the same day after full recovery. The patient was seen in the clinic one week later. He was well with no active issues, and the abdomen was normal on examination.

**Figure 4:**
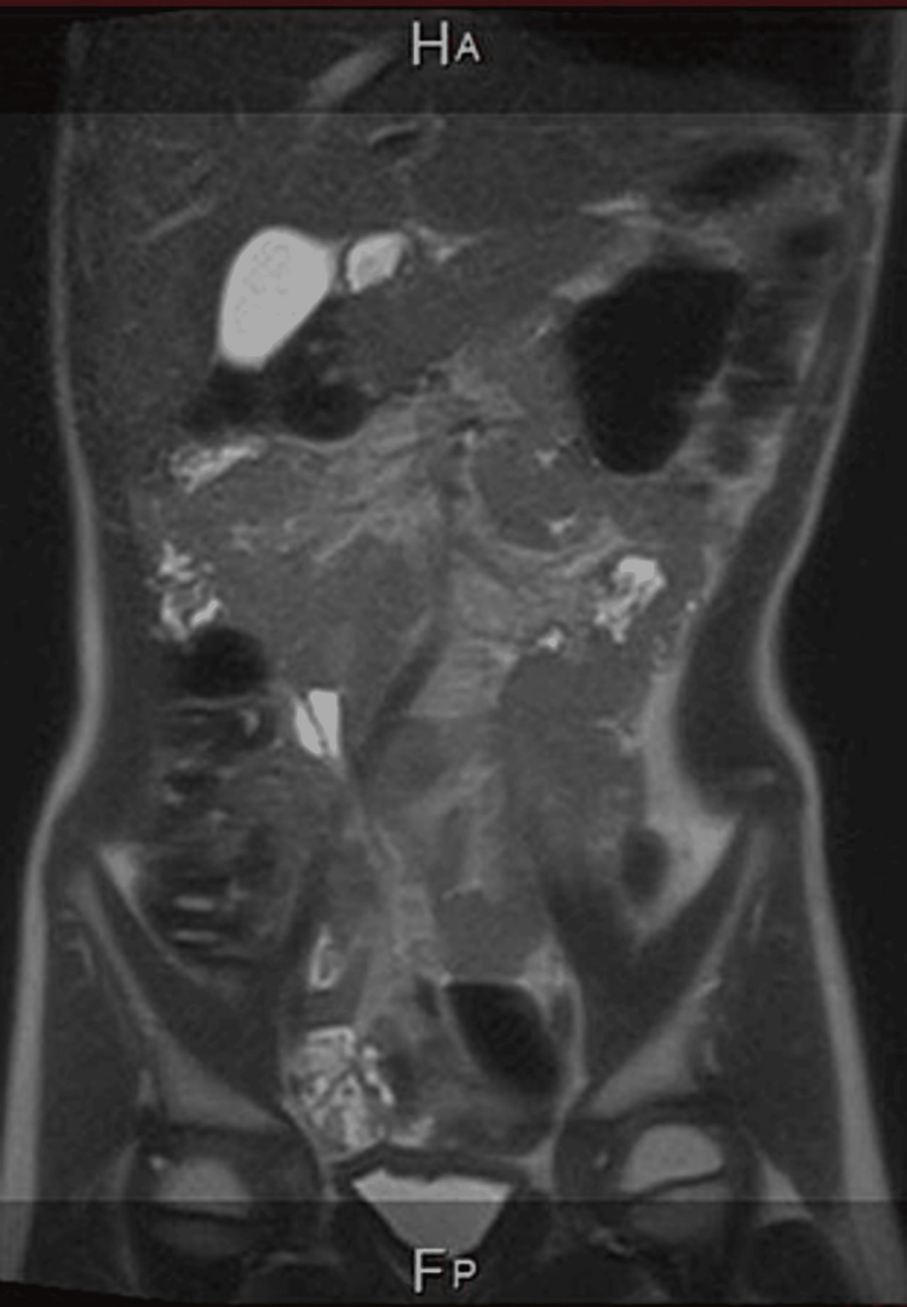
Coronal MRI of the abdomen shows complete resolution of the intra-abdominal lymphangiomas. MRI: Magnetic resonance imaging

Ultrasound of the abdomen done three months after the procedure showed a small cystic structure seen adjacent to the spleen in the left upper quadrant measuring 0.5 cm. No other cystic lesions were identified. A follow-up MRI six months after the sclerotherapy showed complete resolution of the intra-abdominal lymphangiomas (Figure 4). The patient remains under active follow-up, and one year following sclerotherapy he remains asymptomatic.

## Discussion

LMs are benign and usually present at birth or within the first few years of life [1]. They are caused by the defective embryological development of the primordial lymphatic structures and could be classified as macrocystic, microcystic, or mixed cystic lesions [3].

The LM can occur anywhere in the body except in the central nervous system [4]. Up to 75% of LMs are seen in the head and neck but can be also seen in the axilla, mediastinum, groin, and abdomen [1]. Symptoms of LMs vary based on specific anatomical locations and the size of the lesion [1-5].

According to a study conducted on 25 patients with LM in Seoul National University Children’s Hospital from 1991 to 2011, the most common presenting symptoms were abdominal pain in 17 patients, whereas four patients presented with vomiting and four patients with palpable mass [6].

Abdominal ultrasound is an excellent modality for initial evaluation and it is the gold standard to monitor their clinical evolution as it does not require sedation and lacks ionizing radiation. MRI may be used to confirm the diagnosis, and better identify anatomy and relation to other structures.

The current primary treatment modality for macrocystic and combined LMs is percutaneous sclerotherapy [2]. Doxycycline was associated with an overall success rate of 84.2% in treating macrocystic and mixed LMs of the head and neck in children [7]. There is no standard doxycycline dose, and the overall sclerotherapy volume is determined by the intracystic LM volume.

A study conducted on 10 patients with macrocystic LM and one patient with the combined disease showed that in abdominal LM, doxycycline sclerotherapy is helpful for volume reduction and long-term symptom resolution. After therapy, many patients have no residual or noticeable LM [8]. There are minor complications associated with the use of doxycycline in sclerotherapy of LMs. Burrows et al. reported prolonged swelling, pain, and skin blisters. The complications were associated with microcystic or combined lesions and a higher doxycycline concentration [9]. Our patient underwent a single session with multiple injections of doxycycline sclerotherapy without immediate, early, or late complications, and with complete resolution of intra-abdominal LMs after one year of follow-up.

## Conclusions

There is no agreement in the literature regarding the choice of sclerosant in the treatment of LMs. Doxycycline appears one of the choices that promise good results with a low risk of side effects and complications, but a standard dosage or concentration is again lacking in the literature. In our case, doxycycline as a sclerosant and the used concentration were chosen in collaboration with our interventional radiologist to hopefully provide maximum results with minimum complications. Our patient had multiple giant intra-abdominal LMs and responded completely after a single session of sclerotherapy without immediate or late complications. We conclude that doxycycline sclerotherapy is safe and effective in cases of multiple intra-abdominal LMs in children.
